# Neuronal activity-dependent ATP enhances the pro-growth effect of repair Schwann cell extracellular vesicles by increasing their miRNA-21 loading

**DOI:** 10.3389/fncel.2022.943506

**Published:** 2022-09-23

**Authors:** Cristian Saquel, Romina J. Catalan, Rodrigo Lopez-Leal, Ramon A. Ramirez, David Necuñir, Ursula Wyneken, Christophe Lamaze, Felipe A. Court

**Affiliations:** ^1^Center for Integrative Biology, Faculty of Sciences, Universidad Mayor, Santiago, Chile; ^2^Institut Curie, PSL Research University, INSERM U1143, CNRS UMR 3666, Membrane Mechanics and Dynamics of Intracellular Signaling Laboratory, Paris, France; ^3^Geroscience Center for Brain Health and Metabolism, Santiago, Chile; ^4^Facultad de Medicina, Universidad de los Andes, Santiago, Chile; ^5^Center of Interventional Medicine for Precision and Advanced Cellular Therapy, Santiago, Chile; ^6^Buck Institute for Research on Aging, Novato, CA, United States

**Keywords:** Schwann cell, extracellular vesicles, ATP, purinergic receptors, axonal growth, axonal regeneration, miRNA-21

## Abstract

Functional recovery after peripheral nerve injuries is critically dependent on axonal regeneration. Several autonomous and non-cell autonomous processes regulate axonal regeneration, including the activation of a growth-associated transcriptional program in neurons and the reprogramming of differentiated Schwann cells (dSCs) into repair SCs (rSCs), triggering the secretion of neurotrophic factors and the activation of an inflammatory response. Repair Schwann cells also release pro-regenerative extracellular vesicles (EVs), but is still unknown whether EV secretion is regulated non-cell autonomously by the regenerating neuron. Interestingly, it has been described that nerve activity enhances axonal regeneration by increasing the secretion of neurotrophic factors by rSC, but whether this activity modulates pro-regenerative EV secretion by rSC has not yet been explored. Here, we demonstrate that neuronal activity enhances the release of rSC-derived EVs and their transfer to neurons. This effect is mediated by activation of P2Y receptors in SCs after activity-dependent ATP release from sensory neurons. Importantly, activation of P2Y in rSCs also increases the amount of miRNA-21 present in rSC-EVs. Taken together, our results demonstrate that neuron to glia communication by ATP-P2Y signaling regulates the content of SC-derived EVs and their transfer to axons, modulating axonal elongation in a non-cell autonomous manner.

## Introduction

The nervous system function is critically dependent on glial cells, which regulate several neuronal functions. In both the central and peripheral nervous systems (CNS and PNS, respectively), glial cells modulate nerve conduction velocity, provide metabolic support to axons and synapses, and respond to tissue injury by activating mechanisms aimed to restore homeostasis (Jäkel and Dimou, 2017; Barros et al., 2018). In the PNS, regeneration of injured axons depends on the reprogramming of Schwann cells from a differentiated phenotype (dSC) to a repair Schwann cell phenotype (rSC), specialized for clearing myelin debris, recruiting macrophages, and guiding regenerating axons to their targets by secretion of neurotrophic factors and contact-mediated signaling events (Chen et al., 2015; Gomez-Sanchez et al., 2015; Jessen and Mirsky, 2016; Szepesi et al., 2018; Jessen and Arthur-Farraj, 2019). In addition, neuronal responses can be modulated by the horizontal transfer of RNA and proteins by means of extracellular vesicles (Rajendran et al., 2014). Astrocyte-derived extracellular vesicles regulate synaptic activity by miRNA transfer to neurons (Chaudhuri et al., 2018), and microglia-derived extracellular vesicles stimulate neurotransmission (Antonucci et al., 2012). We have demonstrated that rSC-derived exosomes enhance axonal regeneration *in vitro* and *in vivo* (Lopez-Verrilli et al., 2013), an effect dependent on the reprogramming of dSC into a repair phenotype (López-Leal et al., 2020). Exosomes correspond to small extracellular vesicles (EVs) or nano-vesicles (50–120 nm) released after the fusion of multivesicular bodies (MVBs) with the plasma membrane (Théry et al., 2002). During their formation, exosomes are loaded with proteins, lipids, mRNA, and non-coding RNAs (Jeppesen et al., 2019). Exosomes are enriched in specific repertoires of miRNAs, showing an asymmetric distribution when compared to their cells of origin (Valadi et al., 2007; Villarroya-Beltri et al., 2013). Secretion of exosomes is regulated by several mechanisms, including the phenotypic state of the cell, as well as activation by non-cell autonomous mechanisms (Mathieu et al., 2019). In the nervous system, exosome secretion by oligodendrocytes is enhanced by the neurotransmitter glutamate released by neurons (Frühbeis et al., 2013), suggesting that nerve activity might modulate exosome secretion by glial cells. Interestingly, nerve activity enhances axonal regeneration and functional recovery in the PNS (Haastert-Talini and Grothe, 2013). The electrical stimulation of the sciatic nerves of the transected mice increases axonal regeneration across the injured site by 30–50% (Singh et al., 2012), and in a rat model of femoral nerve regeneration, brief periods of electrical stimulation enhance axonal regeneration and muscle reinnervation in a TTX-dependent manner (Al-Majed et al., 2000). Nevertheless, whether neuronal activity modulates the release of pro-regenerative exosomes by rSC after nerve injury has not been investigated.

Using *in vitro* models of neuronal and glial cultures, we demonstrate that sensory neurons activate small EV secretion by rSC through an activity-dependent release of ATP, acting over P2Y receptors in rSCs. Strikingly, activation of this signaling pathway not only enhances the release of rSC EVs but also modifies their miRNA content, increasing the EV expression of the pro-regenerative miRNA-21. Taken together, our results identify a signaling mechanism by which neurons modulate the quantity and quality of glial-derived EVs, enhancing their regenerative capacity.

## Methods

### Schwann cell primary culture

SC primary cultures were obtained from newborn Sprague Dawley (SD) rat sciatic nerves as previously described (Wilby et al., 1999; De Gregorio et al., 2018). Briefly, the perineurium was removed, and the nerve was dissociated in 0.05% trypsin/1% collagenase type I solution. Cells were plated on laminin (40 ng/mL; Millipore)-treated flasks in Dulbecco's Modified Eagle's Medium (DMEM, Invitrogen) supplemented with 10% fetal bovine serum (FBS, Invitrogen) and 10% penicillin-streptomycin (Invitrogen). The following day, cells were treated with 10 mM cytosine arabinoside (Sigma). After 1 week in culture, contaminant fibroblasts were eliminated by complement-mediated cell lysis using an anti-CD90 antibody (Invitrogen) and rabbit complement (Sigma). SCs were maintained in DMEM-10%, FBS-1%, penicillin-streptomycin supplemented with 2 μM forskolin (Millipore), and 20 mg/ml bovine pituitary extract (Invitrogen). SCs were then frozen in 10% DMSO and 90% DMEM and passaged up to five times. For each independent biological replicate, two newborn SD rat sciatic nerves from the same rat were used for the preparation of the cell culture.

### Schwann cell stimuli, EV purification, and analysis

SC primary cultures supplemented with DMEM containing 2 mM forskolin, 20 mg/ml bovine pituitary extract, and 10% EV-free FBS (obtained by serum ultracentrifugation at 100,000 *g* for 2 h) (SC media) were stimulated with different reagents, such as 50 μM ATP (Sigma), 2 μM ionomycin (Sigma), 300 μM suramin, 10 μM BBG, and 100 mM EDTA (Sigma), for 5 min. Then the media was replaced with fresh SC media to eliminate all traces of reagents. The cells were incubated for different time points (1, 3, 6, 8, 16, and 24 h), and the supernatant was subjected to serial centrifugations (2,000 *g* for 10 min and 11,000 *g* for 30 min at 4°C), followed by ultracentrifugation at 100,000 *g* for 60 min at 4°C (T865 rotor, OTD Combi Sorvall ultracentrifuge, Dupont). The pellet containing EVs was washed in cold 0.1 M phosphate buffer saline, pH 7.4 (PBS), and ultracentrifuged again at 100,000 *g* for 60 min at 4°C. In each EV preparation, the concentration of total proteins was quantified by NanoOrange protein quantitation kit (Invitrogen) and stored at −20°C for later use. For the analysis of EVs, the pellets were resuspended in 1 ml of PBS, and the concentration and the particle size distribution were analyzed under a Nanosight NS3000 (Malvern).

### DRG explants culture and axonal elongation assay

Dorsal root ganglia (DRG) were obtained from day 16 SD rat embryos as previously described (López-Leal et al., 2018). Briefly, E16 rat embryos were decapitated, and the limbs and organs were removed. The spinal cord with DRG was dissected and placed on coverslips or glass plates coated with poly-L-lysine (100 ng/mL; Sigma) and collagen (50 μg/mL; Invitrogen). DRG were maintained in Neurobasal medium (Invitrogen) supplemented with 2% B27 (Invitrogen), 2 mM L-glutamine, 50 ng/mL human nerve growth factor (NGF; Invitrogen), and 1% penicillin-streptomycin. In all experiments, DRG were treated with 2.5 μM 5-?uoro-2′-deoxyuridine (Sigma) and 3.75 μM aphidicolin (Sigma) to inhibit the proliferation of SCs by inhibiting the action of DNA polymerase (Spadari et al., 1985; Wallace and Johnson, 1989). Axonal elongation was evaluated by plating DRG explants, and the next day, PBS (control) or EVs from SC in different conditions were added daily for 4 days. Axonal elongation was measured in mm^2^ using the Image J software. For each independent biological replicate, a pool of 12–15 SD rat embryos was used for the preparation of the DRG cell culture.

### mRNA and miRNA qPCR

The expression levels of mRNA were evaluated by plating DRG explants, PBS (control), or EVs from SC in different conditions, and were added daily for 4 days. Then, the pool of DRG was homogenized in TRIzol reagent (Life technologies) for the extraction of total RNA by the Trizol method. The aqueous phase was precipitated with 1 volume of isopropanol, 0.1 volumes of 3 M sodium acetate, and 10 μg of glycogen overnight at −20°C. cDNA synthesis was performed with 5x iScript Reverse Transcription Supermix (BioRad). RNA levels of each sample were evaluated by real-time PCR using 5x HOT FIREpol Evagreen qPCR Mix Plus (Medibena). The mixture was run in a real-time PCR thermal cycler (CFX96 Touch Real-Time PCR Detection System, BioRad). Thermal cycling parameters were 15 min at 95°C, followed by 40 cycles of 15 s at 95°C, 20 s at 60°C, and 20 s at 72°C. At the end of the program, melting curve analysis was performed from 60 to 95°C. The following primers were used: *PTEN:* Forward AAGGACGGACTGGTGTAA and Reverse CCTGAGTTGGAGGAGTAGAT, *SPRTY2:* Forward GCAGGATACGCGCTTGGG and Reverse CCTCACAGCGGCTCAACTC, *TIMP3:* Forward CTGGAGCCTTGGGCACTG and Reverse CGGATCACGATGTCGGAGT, and *GAPDH:* Forward TCCCTCAAGATTGTCAGCAA and Reverse AGATCCACAACGGATACATT. For relative comparison, the Ct value was analyzed with the ΔΔCt method of normalizing.

The miRNA levels of miRNA-21 were analyzed in SCs, and their EVs were treated with or without 50 μM ATP. SC and SC-derived EVs were homogenized in Qiazol reagent (Qiagen) for the extraction of total RNA based on the miRNeasy Mini kit (Qiagen) protocol. MicroRNA qPCR for miRNA-21 was performed using the MystiCq MicroRNA Quantitation System (Sigma) according to the manufacturer's protocol; briefly, miRNA cDNA of 20 ng of RNA was synthesized using the MystiCq microRNA cDNA synthesis mix (Qiagen). The miRNA levels of each sample were determined by real-time PCR using MystiCq microRNA SYBR Green qPCR ReadyMix (Qiagen). The mixture was run in a real-time PCR thermal cycler (Lightcycler System; Roche Diagnosis Corp). Thermal cycling parameters were 120 s at 95°C, followed by 40 cycles of 5 s at 95°C, and 30 s at 60°C. At the end of the program, a melting curve analysis was performed at 60°C for 30 s, followed by a cooling step at 37°C for 30 s. Each sample was also run with primers for a Cel-miR39 spike-in RNA (Qiagen) introduced in the cDNA synthesis step. Primers for rat miRNA-21 (rno-miR-21-5p; MIRAP00047) were ordered from Sigma. For relative comparison of miRNA-21 levels, the Ct value was analyzed with the ΔΔCt method of normalizing.

### Functional assays

For the gain and loss of function assays, we used miRNA mimics and inhibitors (Exiqon) for miRNA-21 from *Rattus norvegicus*. First, DRG explants were transfected with lipofectamine 2000 (Thermo Fisher) with the miRNA-21 mimic 1 day after plating the DRG, and axonal elongation was quantified 4 days post-transfection. For the loss-of-function assay, SCs were transfected with lipofectamine 2000 with 50 nM LNA-enhanced antisense miRNA inhibitor of miRNA-21 or a negative control A inhibitor (YI00199006) (Exiqon) in Optimem media (Gibco). The media was replaced after 4 h with SC media. Then, EVs were purified by ultracentrifugation from SCs incubated with or without 50 μM ATP after 8 h, and DRG explants were treated daily with 0.5 μg of these EVs. Axonal elongation was measured on day 4 after treatment.

### Extracellular vesicle internalization assay

SCs were transduced with a lentivirus coding for CMV-Palm-eGFP (Lai et al., 2015). EVs were purified from SC incubated with or without 50 μM ATP for 8 h by differential ultracentrifugation and quantified by NanoOrange protein assay. DRG were treated with 5 μg of EVs and were fixed 4 h later.

### Co-culture of schwann cells and sensory neurons

SCs were transduced with a lentivirus coding for CMV-Palm-eGFP (Lai et al., 2015). Twenty-four hours after the transduction process, SCs were trypsinized and seeded over DRG plated 7 days before and left for 3 days before the experiments were performed. Co-cultured cells were treated with apyrase 1.5 U/mL (Sigma) or 0.5 μM TTX, and the EV release from the SC and internalization by the axon were measured in a Leica TCS SP8 spectral confocal microscope, measuring the colocalization of the GFP-tagged EVs with the axon NFM signal.

### Immunofluorescence

The culture medium was removed from SC or DRG cultures, washed three times with PBS, and fixed with 4% PFA for 15 min at RT. Blocking and permeabilization were done with 0.1% Triton X-100 and 2% fish skin gelatin in PBS for 2 h at RT. Then, the cells were incubated with the primary antibody in 0.1% Triton X-100 and 1% fish skin gelatin in PBS and incubated overnight at 4°C. Then, the primary antibody was removed, the cells were washed three times with PBS, and the secondary antibody diluted in 0.1% Triton X-100 and 1% fish skin gelatin in PBS was incubated for 2 h at RT. Afterward, the secondary antibody was removed, and the cells were washed two times with PBS and one time with DAPI stain in PBS (100 ng/ml). Then, the cells were washed once more in PBS, and coverslips were mounted with fluoromount-G (EMS).

### Statistical analysis

All data are presented as mean ± SEM from the indicated number of experiments. Statistical analysis was performed using Student's *t*-test, linear regression, or ANOVA with Tukey's multiple comparison tests.

## Results

### Neuronal activity increases extracellular vesicle release by rSC and their transfer to axons in an ATP-dependent manner

**Figure 1:**
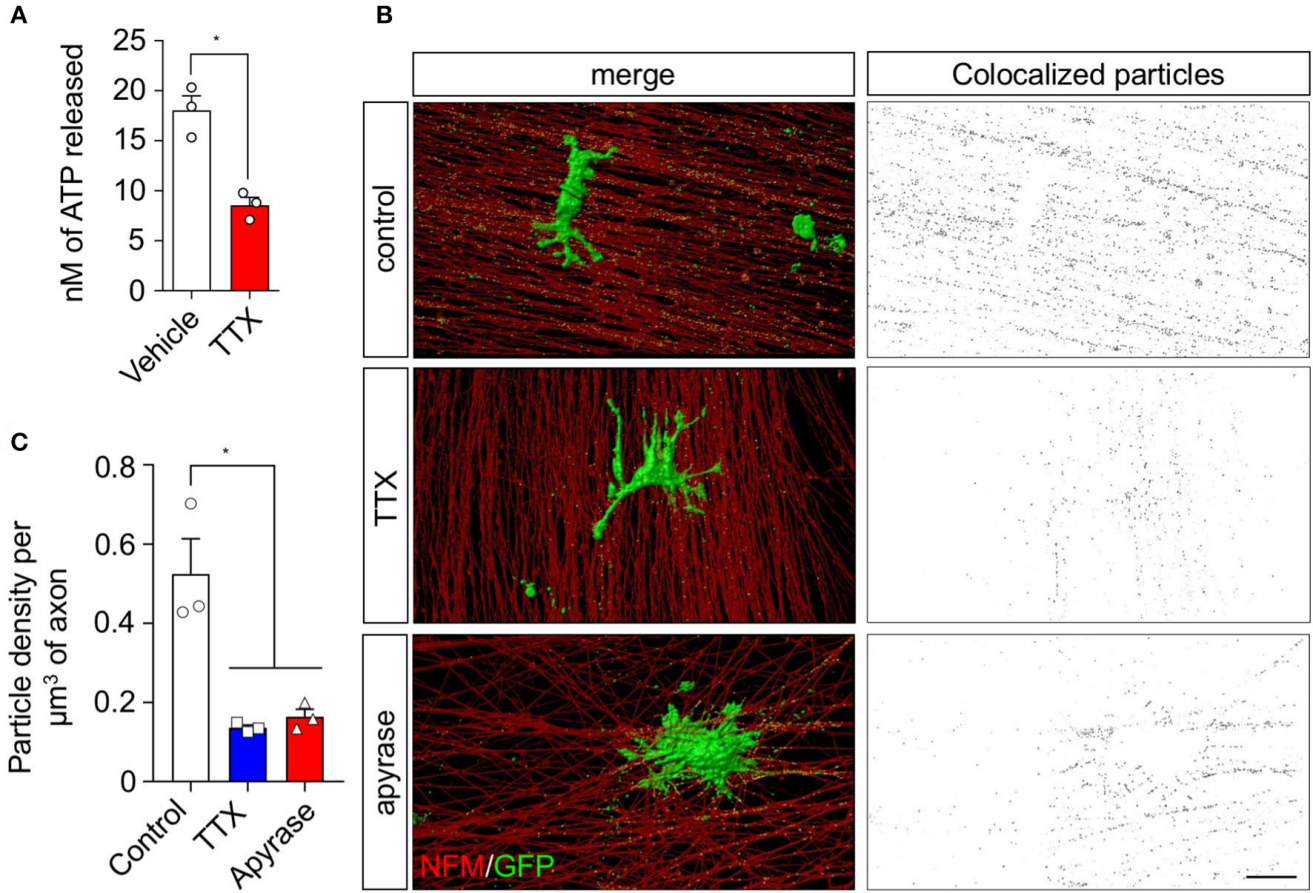
sEV transfer from rSC to axons in co-culture is inhibited upon ATP depletion and action potential inhibition. The involvement of neuronal ATP and spontaneous activity in the transfer of EVs from rSC to axons was analyzed in a co-culture system. **(A)** DRG neurons from primary culture were seeded in white bottom culture plates and left to grow for 7 days; ATP release from neurons was analyzed by luminescence with the ATPlite detection kit in a luminescence spectrometer. **(B)** DRG neurons co-cultured with rSC expressing CD63-GFP to visualize EVs were analyzed for EV transfer by immunofluorescence and detection of GFP signal in axons. Scale bar, 20 μm. **(C)** Colocalized particles were quantified as described in Supplementary Figure 1 and plotted as particle density per volume of axons. In all quantifications, the average and SEM values of at least three independent experiments are shown (*p < 0.05).

As activity-dependent ATP secretion by peripheral neurons has been associated with the activation of signaling pathways in SCs (Negro et al., 2016; Rodella et al., 2017), we investigated the effect of nerve activity on small EV release by SCs and their transfer to axons. To this end, we used sensory neurons from dorsal root ganglia (DRG), which have been previously demonstrated to have spontaneous activity *in vitro* (Matsuka et al., 2008; Mutafova-Yambolieva and Durnin, 2014). Indeed, purified DRG neurons *in vitro* release ATP to the culture medium, which is decreased more than 2-fold after blocking action potentials using the voltage-dependent sodium channel blocker tetrodotoxin (TTX, Figure 1A). We then evaluated whether this TTX-dependent ATP secretion induces a change in the release and uptake of SC-derived EVs. rSCs expressing GFP-labeled EVs were seeded over DRG neurons, and the GFP signal associated with axons was analyzed using a custom-developed particle detection method from confocal-derived images (Supplementary Figure 1 and Methods section). In neurons co-cultured with rSC for 72 h, extensive GFP-positive puncta colocalized with axons immunolabeled against neurofilament protein (Figures 1B,C). After blocking neuronal activity using TTX, rSC-derived GFP signal associated with axons decreases by several folds (Figures 1B,C), and hydrolyzing extracellular ATP using apyrase diminishes the amount of rSC-derived EVs in axons by a magnitude similar to that of TTX (Figures 1B,C). Importantly, TTX or apyrase does not inhibit EV release by rSC (Supplementary Figure 2), suggesting that spontaneous neuronal activity enhances EV release by rSC through a mechanism associated with activity-dependent ATP release.

### ATP enhances the pro-growth capacities of rSC-derived extracellular vesicles

**Figure 2:**
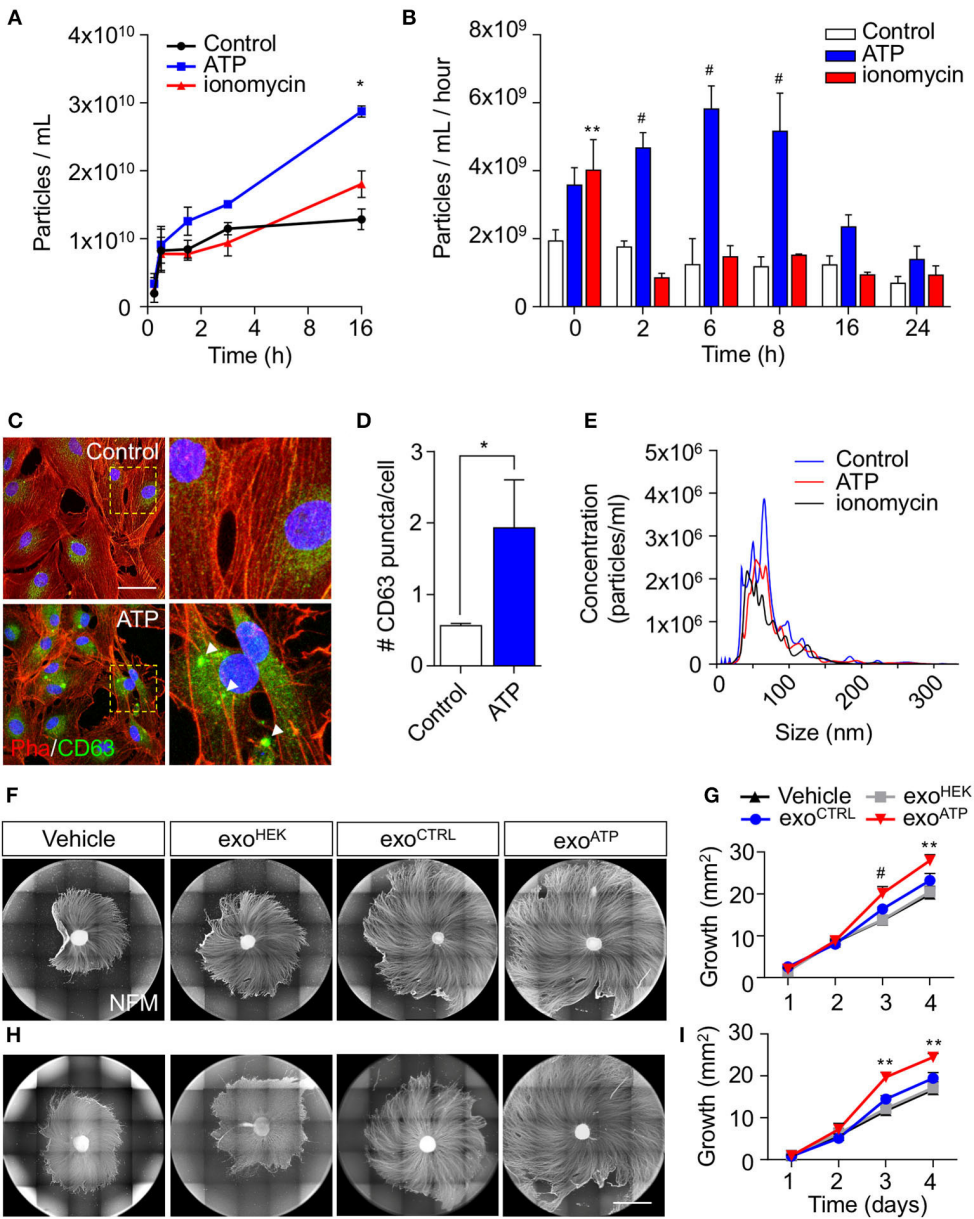
Effect of ATP in the release of sEVs from rSC and their effect on axonal elongation. The effect of ATP (50 μM) on rSC sEV was analyzed by nanoparticle tracking analysis (NTA) and immunofluorescence. **(A,B)** Conditioned media from different time points after ATP treatment were measured by NTA to analyze the accumulation of particles over time and their rate of release by rSC. **(C,D)** CD63-transduced rSC were quantified for GFP puncta (arrowheads) after ATP treatment. **(E)** Size distribution of EVs in the different conditions generated by NTA. **(F,G)** The effect of sEV from rSC treated with or without ATP (50 μM) on neurons was analyzed by daily treatment of DRG with these sEVs. sEVs from the same volume of conditioned media from rSC stimulated with or without ATP were used to treat DRG daily. **(H,I)** sEVs normalized by quantity to 0.5 μg of protein from rSC stimulated with or without ATP were used to treat DRG daily. The elongation effect on DRG was visualized on day 4 by immunofluorescence using neurofilament medium (NFM) immunostaining in axons. Scale bar, 1,500 μm. In all quantifications, average and SEM of at least three independent experiments are shown (^#^*p* < 0,0001; **p < 0.001; *p < 0.05).

As rSC-derived EV secretion can be modulated by activity-dependent ATP release from sensory neurons, we next used purified rSC in order to study the dynamics of this phenomenon and their effect on axonal growth. To this end, rSCs were stimulated with ATP, and the number of released EVs was quantified using nanoparticle tracking analysis (NTA) after differential ultracentrifugation of the conditioned media (see Methods section). In control rSC cultures, EV content in the culture medium reaches a plateau after 3 h. In contrast, EVs from ATP-stimulated rSCs continue to accumulate after 3 h, reaching a 3-fold increase compared to control at 16 h post-stimulation (Figure 2A). Ionomycin, which transiently increases intracellular Ca^2+^ in rSC (not shown), has no noticeable effect on EV secretion (Figure 2A). We then study if this ATP-dependent increase in EV accumulation is associated with changes in the rate of EV release by rSC. For this, rSCs were treated with vehicle, ATP, or ionomycin, and 1-h conditioned media was collected at different time points post-stimulation. As expected, in control conditions, the rate of EV release was constant for up to 24 h, and stimulation with ionomycin led to an increase in EV release only in the first hour (Figure 2B), which probably corresponds to the ready-releasable pool of MVBs present in rSCs. Remarkably, an acute 5-min ATP treatment leads to a long-lasting 3-fold increase in the rate of EVs released by rSC until 8 h post-treatment (Figure 2B). Associated with this increase in EV release, rSC at 8 h post-ATP treatment showed an increase in the presence of enlarged clusters of the MVB marker CD63 compared to vehicle-treated cells (Figures 2C,D). Importantly, changes in the rSC EV secretion rate triggered by ATP were not accompanied by major alterations in the EV size distribution as revealed by NTA (Figure 2E).

As we have previously demonstrated that rSC-derived exosomes increase axonal growth and regeneration of sensory neurons (Lopez-Verrilli et al., 2013), we tested the pro-growth effect of EVs obtained from control and ATP-stimulated rSC. We first tested the effect using EVs secreted from equal volumes of conditioned media of control and ATP-treated rSC. DRG devoid of SC were daily treated with vehicle, EVs from control, or ATP-treated rSC, and the axonal extension was evaluated (Figure 2F). EVs derived from HEK cells were used as an additional control. As expected, control rSC-derived EVs enhanced axonal regeneration when compared to vehicle or HEK-derived EVs (Figure 2F). Interestingly, EVs derived from ATP-rSC showed an even higher pro-regenerative effect (Figures 2F,G). Since ATP-treated rSCs released a higher number of EVs (Figures 2A,B), we performed the same assays using comparable amounts of EVs for the different conditions (assessed by protein content or EV number analyzed by NTA, see Supplementary Table 1). Remarkably, when normalized, ATP-treated rSC-derived EVs still possess a higher pro-growth capacity than control rSC or HEK-derived EVs (Figures 2H,I). Importantly, EVs from control or ATP-treated rSC were similarly internalized by DRG axons (Supplementary Figure 3). Taken together, these results demonstrate that ATP enhances the rate of EV release by rSCs and modifies the pro-growth capacity of these EVs.

### ATP-dependent enhancement of the pro-growth capacity of rSC extracellular vesicles is associated with the activation of P2Y receptors in SCs

**Figure 3:**
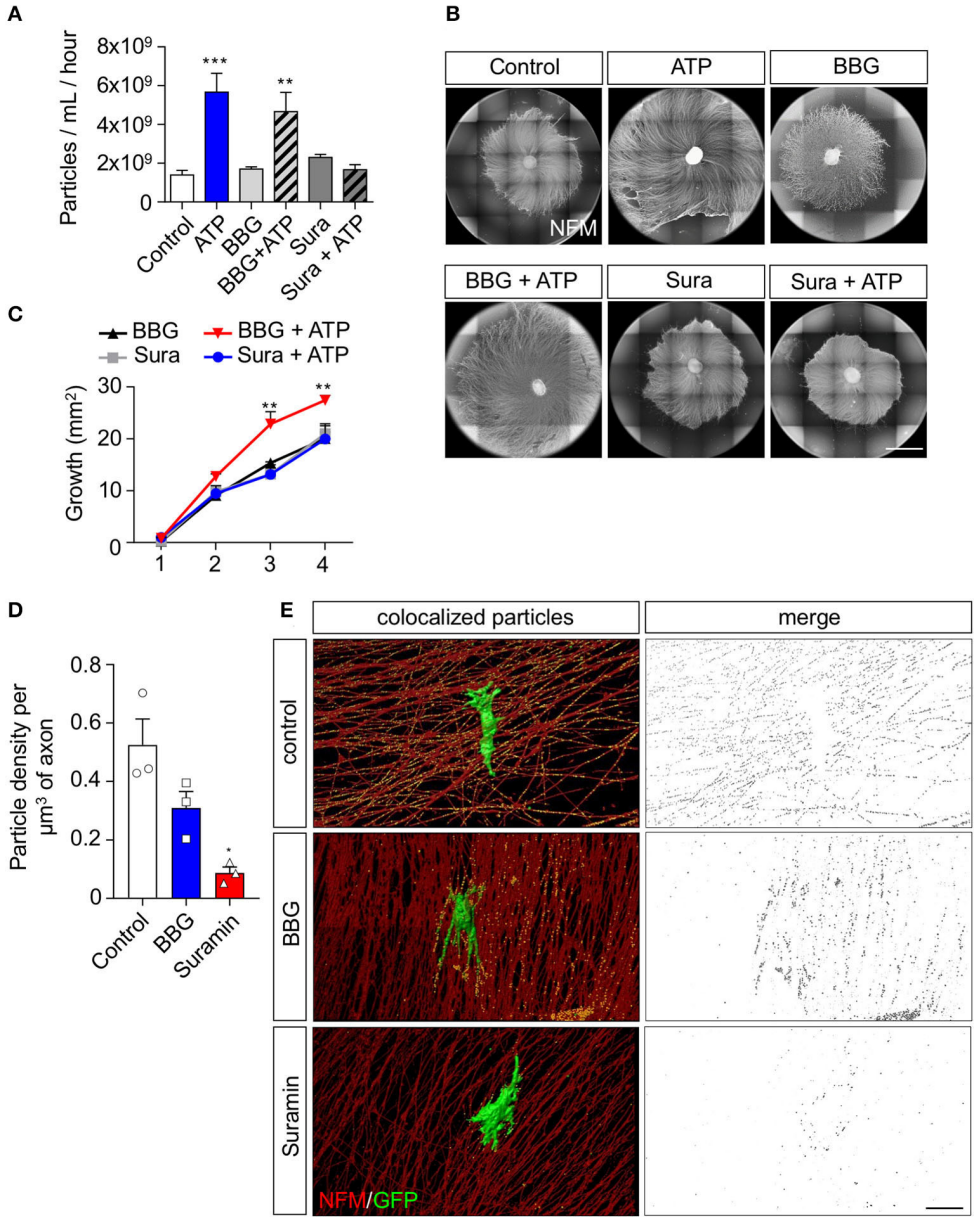
P2Y family of receptors is involved in the increased sEV release and axonal elongation effect of ATP on rSC. To analyze to which receptor ATP binds to exert its effect on rSC we pharmacologically inhibited P2X7 or P2Y receptors and analyzed the sEV released by NTA and their effect on axonal growth in DRG after ATP treatment. **(A)** Conditioned media from 8 h after ATP and drug treatment were measured by NTA to analyze the rate of release of sEV from rSC. **(B,C)** The effect of sEV from rSC treated with or without ATP in the presence of BBG or suramin on neurons was analyzed by daily treatment of DRG with 0.5 μg of sEV. The elongation effect on DRG was visualized on day 4 by immunofluorescence using neurofilament medium (NFM) immunostaining in axons. Scale bar, 1,500 μm. **(D,E)** Co-cultured DRG neurons with rSC expressing CD63-GFP to visualize EVs were analyzed for EV transfer by immunofluorescence after incubation with BBG or suramin and detection of GFP signal in axons. Scale bar, 20 μm. In all quantifications, average and SEM of at least three independent experiments are shown (***p < 0,0001; **p < 0.001; *p < 0.05).

Extracellular ATP can activate metabotropic or ionotropic purinergic receptors, both expressed by SCs (del Puerto et al., 2013). Therefore, we used a pharmacological strategy to evaluate the pathway by which ATP enhances rSC-EV secretion rates and their pro-growth properties. First, we analyzed the effect of the P2X7 and P2Y receptor antagonists BBG and suramin, respectively, on EV release rate. Although both BBG and suramin do not change the basal release rate of rSC-EVs, the ATP-induced increase of EV release was completely abrogated by suramin (Figure 3A). Next, we analyzed the effect of purinergic receptor inhibition on the pro-growth effect of EVs secreted by ATP-treated rSC. Indeed, EVs from suramin-treated ATP-rSC lose their pro-growth activity over sensory neurons (Figures 3B,C). Together, these data show that ATP activates P2Y metabotropic receptors in Schwann cells, leading to an increase in the rate of EV release, as well as an enhancement in their pro-growth effect over peripheral neurons. Considering the inhibitory effect of suramin over the rate of EV secretion and their pro-growth capacity in ATP-treated rSC, we next evaluated the effect of purinergic inhibition in a co-culture system as described above. In agreement with the previous results, suramin strongly inhibited the transfer of GFP-labeled EVs from SCs (Figures 3D,E). Taken together, these results support our hypothesis that neuron-derived ATP released under spontaneous activity acts through P2Y receptors in rSC, and not P2X, to induce the release and transfer of EVs from rSC to axons.

### The enhanced growth effect of extracellular vesicles from ATP-activated rSC depends on the increased expression of miRNA-21

**Figure 4:**
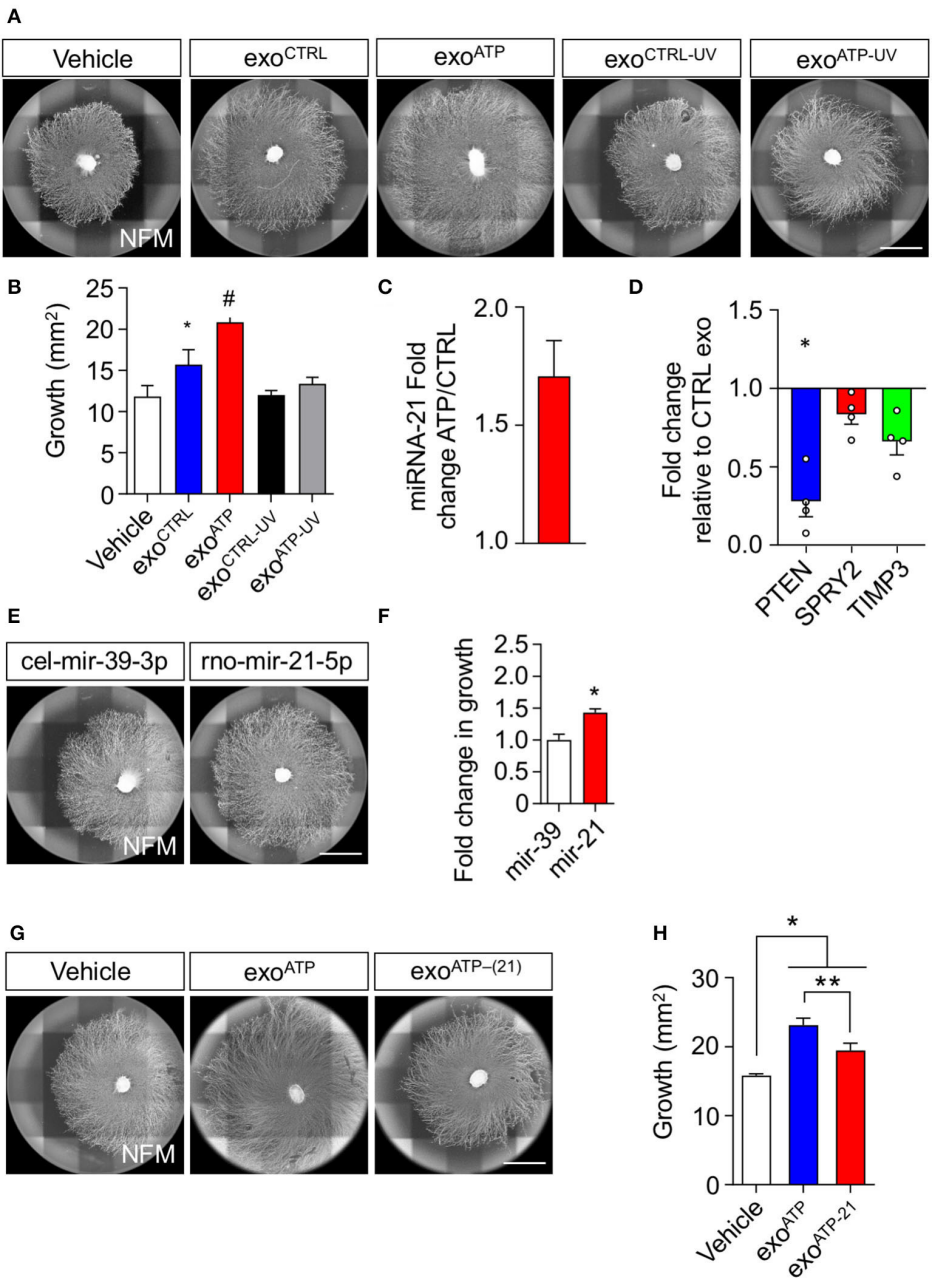
miRNA-21 is a key participant in the axonal elongation effect of rSC-derived sEV on DRG neurons. **(A,B)** RNA participation in the axonal elongation effect of sEV over DRG neurons was first evaluated by UV irradiation. Axonal growth was analyzed on day 4 after adding rSC-derived sEV daily from SC treated with or without ATP to the DRG neurons. For RNA inactivation, EVs were previously UV irradiated. The axonal elongation of DRG induced by sEV was visualized by immunofluorescence using neurofilament medium (NFM) immunostaining in axons. Scale bar, 1,500 μm. **(C)** miRNA-21 content in rSC-derived sEVs was assessed by qPCR in sEV samples from control or ATP-stimulated rSCs. **(D)** Expression of PTEN, SPRY2, and TIMP3 was measured by qPCR from DRG neurons after treatment with sEV derived from control or ATP-treated rSC. **(E,F)** The involvement of miRNA-21 was assessed by transfection of miRNA-21 inhibitors or mimics in rSC. miRNA-21 was directly transfected in DRG neurons, and the axonal growth area was analyzed 4 days after transfection, and the miRNA mimic mir-39-3p with no known rat mRNA target was used as a control. The increased axonal growth induced by transfection of miRNA-21 was visualized by immunofluorescence using neurofilament medium (NFM) immunostaining in axons. Scale bar, 1,500 μm. **(G,H)** SCs were transfected with miRNA-21 inhibitors, sEVs were purified, DRG were treated daily with these sEVs, and the axonal growth area was analyzed 4 days after transfection. The effect of the inhibitors on the sEVs and their axonal elongation effect were visualized by immunofluorescence using neurofilament medium (NFM) immunostaining in axons. Scale bar: 1,500 μm. In all quantifications, average and SEM of at least three independent experiments are shown (^#^*p* < 0,0001; **p < 0.001; *p < 0.05).

We have already demonstrated that miRNA contained in rSC exosomes enhances axonal growth (López-Leal et al., 2020). Therefore, we hypothesized that miRNAs could be implicated in the pro-growth effect of ATP-treated SCs. We first used ultraviolet (UV) treatment to photochemically damage RNA molecules contained in rSC EVs (Eldh et al., 2010; Zhang et al., 2016). We observed that UV exposure completely abolished the increase in axonal growth of ATP-rSC-derived EVs (Figures 4A,B). We also previously demonstrated that miRNA-21 contained in rSC exosomes mediates their pro-growth effect over sensory neurons (López-Leal et al., 2020). Importantly, miRNA-21 contained in rSC-EVs showed a 1.7-fold increase in ATP-treated rSC compared to non-treated rSC (Figure 4C). miRNA-21 targets various genes involved in the regulation of neurite elongation and axonal growth, such as PTEN, SPRY2, and TIMP3 (Duraikannu et al., 2019; Jamsuwan et al., 2020; Borger et al., 2022); therefore, we evaluated whether ATP-rSC-derived EVs regulate the expression of these genes in DRG neurons. Indeed, we observed a 70% decrease in the expression of PTEN, a 15% decrease in the expression of SPRY2, and a 30% decrease in the expression of TIMP3 in neurons treated with ATP-rSC-derived EVs compared to neurons treated with control rSC-derived EVs (Figure 4D). To evaluate the participation of miRNA-21 in axonal growth, we first transfected DRG neurons with a miRNA-21 oligonucleotide mimic. Indeed, miRNA-21 overexpression in DRG increases axonal growth when compared to the non-targeting miRNA-39-3p (Figures 4E,F). We next assessed the participation of rSC miRNA-21-containing EVs in the elongation of axons. For this, a miRNA-21 inhibitor was transfected into rSCs, and secreted EVs were used for growth assays. Depleting miRNA-21 from ATP-stimulated rSCs causes a decrease in growth enhancement provided by the secreted EVs (Figures 4G,H). In summary, these results demonstrate that ATP increases the presence of miRNA-21 in rSC-derived EVs, which is responsible for their pro-growth effect on DRG neurons.

## Discussion

The role of EVs in intercellular communication and delivery of active molecules has been widely described in the nervous system (Lopez-Verrilli et al., 2013; Prada et al., 2018). Furthermore, exosome release from glial cells has been linked to synaptic activity and neurotransmitter release from neurons in the central nervous system (Frühbeis et al., 2013). In this work, we have been able to thoroughly characterize the mechanism by which neurons are able, through the release of ATP, to signal SCs into modifying their sEV dynamics by not only increasing their secretion rate but also changing their miRNA cargo into a specific phenotype that is beneficial for neurons and their neurite growth.

Our previous studies demonstrated that SC-derived exosomes induce an increase in the axonal elongation of sensory neurons (Lopez-Verrilli et al., 2013), but the mechanism that causes this increased neurite growth remained a mystery. Recent studies have shown that ATP is released by degenerating cerebellar granular neurons and spinal cord motor neurons. Moreover, ATP is capable of triggering Ca^2+^ spikes, cyclic AMP production, and activating the ERK/CREB pathway in SC co-cultured with these neurons (Negro et al., 2016; Rodella et al., 2017). It is important to note that SCs express ionotropic and metabotropic purinergic ATP receptors, including P2X7, P2Y1, and P2Y2 (Fields and Stevens, 2000). The involvement of these purinoreceptors in EV dynamics has been described in various models. It has been shown that the activation of the P2X7 receptor is able to induce the release of large and small EVs in microglia (Bianco et al., 2009; Asai et al., 2015). Furthermore, the microglial-EVs released upon ATP stimulation have been associated with various phenotypes in neurological diseases, such as traumatic brain injury or multiple sclerosis (Matute et al., 2007; Verderio et al., 2012). On the other hand, the P2Y family of receptors has been described to have a positive role in platelet-EV formation and release, in a multi-step process involving an increase in intracellular calcium concentration, decrease in intracellular cAMP concentration, and activation of a signaling cascade including PI3K, which leads to the activation and phosphorylation of various target proteins that eventually lead to the increased platelet-EV formation (Gasecka et al., 2020). Interestingly, we show that the increased release of SC-derived EVs induced by ATP is not dependent on P2X receptors but rather dependent on the P2Y family of receptors and lasts for several hours, which suggest that there is a long-lasting effect, consistent with the activation of intracellular pathways.

Changes in the RNA cargo of EVs have been reported in several cell types after induction by certain stimuli. Cardiomyocytes subjected to ischemia change the miRNA cargo of their EVs promoting cardiac angiogenesis (Ribeiro-rodrigues et al., 2017). TNFα and IL-1β have been shown to modify a specific set of miRNAs in EVs derived from astrocytes to diminish the activity of target neurons (Chaudhuri et al., 2018). To date, only a handful of studies have demonstrated that ATP is able to impact EV composition. It has been shown that microglia-derived EVs present a distinct protein content after a prolonged ATP exposure compared to EVs from non-stimulated cells (Drago et al., 2017). Furthermore, EVs from ATP-stimulated microglia have a stronger impact on the activation state of recipient cells (Drago et al., 2017).

Even though the mechanism by which ATP, through P2Y receptors, leads to a change in the composition of EV miRNAs remains to be explored, different sorting mechanisms for the asymmetric miRNA expression in EVs have been described, including those controlled by the recognition of specific miRNA motifs by loading proteins, as well as regulation by the cellular levels of the target transcripts (Villarroya-Beltri et al., 2013; Santangelo et al., 2016).

Among the miRNAs that are more abundant in ATP-stimulated SC-derived EVs, we found miRNA-21. miRNAs have already been shown to be involved in the regulation of neuron growth. During the early stages of brain development, miRNA-29c is expressed and promotes neurite outgrowth by decreasing PTEN expression (Zou et al., 2015). We have previously described that SC reprogramming into repair cells modifies the exosomal cargo, promoting neurite growth in receiving neurons. Furthermore, this effect is dependent on the selective loading of miRNA-21 into the secreted exosomes (López-Leal et al., 2020). Interestingly, PTEN and SPRY2 are mRNA targets of miRNA-21, and DRG treated with EVs derived from ATP-stimulated SC show a decrease in the levels of PTEN and SPRY2. We hypothesize that this decrease in PTEN and SPRY2 expression may be partially responsible for the axonal elongation effect seen on EV-treated DRG. Interestingly, this effect has already been reported in the growth of sensory neurons *in vitro* and *in vivo* regeneration after spinal cord injury, where miRNA-21 promotes neurite outgrowth by regulating PTEN and SPRY2 (Jiang et al., 2017; Jamsuwan et al., 2020).

It has been demonstrated that nerve stimulation activates pro-regenerating programs in both neurons and glial cells (Willand et al., 2016), including an increase in the secondary messenger cAMP in peripheral neurons (Aglah et al., 2008; Udina et al., 2008) and the upregulation of neurotrophic factors by neurons and SCs (Al-Majed et al., 2000; Huang et al., 2010; Sharma et al., 2010; Wan et al., 2010). In the PNS, neurons release ATP during the firing of action potentials, and after nerve injury, this ATP then activates purinergic receptors in SCs, which lead to Ca^2+^ spikes in these cells and cause the activation of ERK/CREB, a signaling pathway with a key role in peripheral nerve regeneration (Negro et al., 2016). Our work establishes an unexpected mechanism by which neurons can modulate axonal elongation in a non-cell autonomous fashion, which involves the modulation of the miRNA cargo of SC-derived EVs. Overall, our data provide evidence about new checkpoints that could serve to develop strategies to enhance axonal elongation in conditions where regeneration is greatly impaired.

## Data availability statement

The original contributions presented in the study are included in the article/Supplementary material, further inquiries can be directed to the corresponding author.

## Ethics statement

The animal study was reviewed and approved by CBB, Universidad Mayor, Santiago, Chile.

## Author contributions

CS: planning and executing experiments and writing the manuscript. RC, RL-L, RR, and DN: planning and executing experiments. UW, CL, and FC: planning experiments. All authors contributed to the article, reviewed the final manuscript and approved the submitted version.

## Funding

This work was supported by grants from ECOS-ANID N°C17S03, Geroscience Center for Brain Health and Metabolism, FONDAP N°15150012, and Fondo Nacional de Desarrollo Científico y Tecnológico (FONDECYT) N°1150766 (to FC).

## Conflict of interest

The authors declare that the research was conducted in the absence of any commercial or financial relationships that could be construed as a potential conflict of interest.

## Publisher's note

All claims expressed in this article are solely those of the authors and do not necessarily represent those of their affiliated organizations, or those of the publisher, the editors and the reviewers. Any product that may be evaluated in this article, or claim that may be made by its manufacturer, is not guaranteed or endorsed by the publisher.

## Supplementary material

Supplementary Figure 1 Particle transfer detection protocol. **(A)** Images were taken using a size of 1,024 × 512 pixels, and each stack was taken every 0.2 μm with a speed of 200 Hz. Background levels were normalized using the software LAS X based on the control image corresponding to not infected (GFP-palm) SC and DRG co-cultures incubated with the primary anti-GFP and secondary antibody Alexa 488. The value of filter settings was 7, which allowed the total subtraction of the non-specific signal. Each image was deconvolved in 3D in the software LASX using a total of 10 iterations, under the blind method, and then the images were analyzed using IMARIS 9.2.1 software. Scale bar, 25 μm. **(B)** For each channel, a surface was created using the voxel number algorithm. The SC surface reconstruction (green channel) was performed with a detail of 0.4 μm and a threshold setting of 0.5, and subsequently, a mask that contains the voxels outside of this surface was used for analysis. The axon surface analysis (red channel) was performed with a detail of 0.2 μm and a threshold setting of 5, and a mask of this was created for analysis. The voxels outside the surface mask of the green channel were colocalized with the surface of the red channel mask, adjusting the threshold to a value of 2 for all images. The colocalization mask was analyzed with the spot detection tool, detecting particles between 80 and 400 nm. The particle density was determined by taking the number of spots and dividing for the axonal volume. Scale bar, 20 μm.

Supplementary Figure 2 TTX and apyrase treatments do not affect exosome release from rSC. rSCs were treated with ATP, TTX, and/or apyrase, and the conditioned media from each condition was centrifuged to purify sEVs. Samples were measured by NTA to analyze the release of particles in 1 h. In all quantifications, average and SEM values of at least three independent experiments are shown (^*^*p* < 0.05).

Supplementary Figure 3 Internalization of rSC exosomes by DRG neurons. Exosomes were isolated from control or ATP-treated rSCs transfected with CMV-palm-eGFP to visualize EVs. **(A)** DRG explants were treated with 5 μg of EVs for 3 h, washed, and immunostained against neurofilament heavy chain (NF-H, red) and GFP (green). Scale bar, 2 μm. **(B)** The axon-exosome colocalization percentage was obtained from deconvolved z-stack confocal images by measuring the eGFP mean staining area colocalized with the NF-H staining. Scale bar, 15 μm. In all quantifications, average and SEM values of at least three independent experiments are shown.

Supplementary Table 1 Protein quantification by NanoOrange and particle count by Nanosight.
