# Supplementary figures and images for: Neuronal activity-dependent ATP enhances the pro-growth effect of repair Schwann cell extracellular vesicles by increasing their miRNA-21 loading
Source: Front Cell Neurosci. 2022 Sep 23;16:943506. doi: 10.3389/fncel.2022.943506 (PMC9537768; doi:10.3389/fncel.2022.943506)

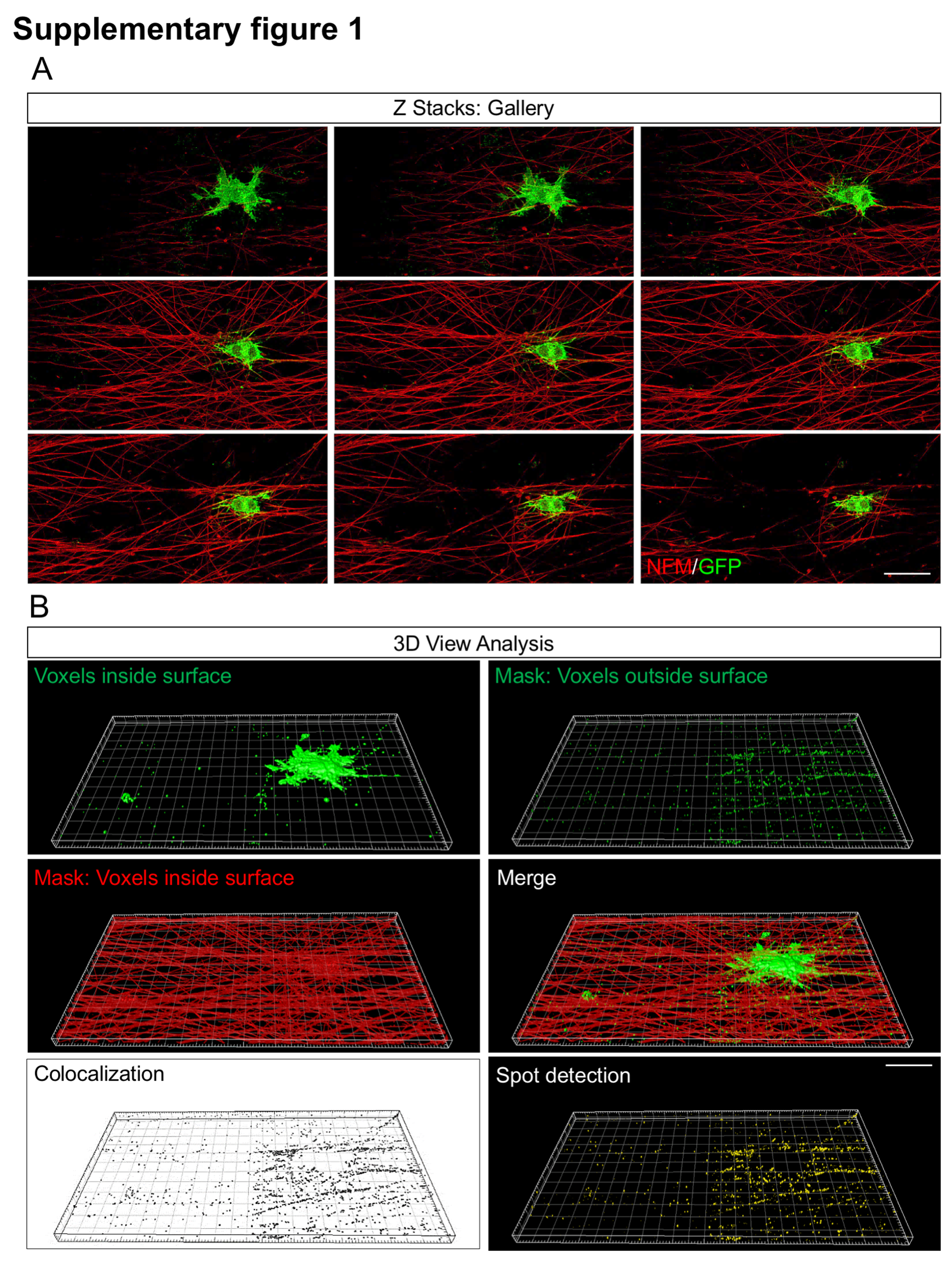

Supplement: Supplementary Figure 1 — Particle transfer detection protocol. (A) Images were taken using a size of 1,024 × 512 pixels, and each stack was taken every 0.2 μm with a speed of 200 Hz. Background levels were normalized using the software LAS X based on the control image corresponding to not infected (GFP-palm) SC and DRG co-cultures incubated with the primary anti-GFP and secondary antibody Alexa 488. The value of filter settings was 7, which allowed the total subtraction of the non-specific signal. Each image was deconvolved in 3D in the software LASX using a total of 10 iterations, under the blind method, and then the images were analyzed using IMARIS 9.2.1 software. Scale bar, 25 μm. (B) For each channel, a surface was created using the voxel number algorithm. The SC surface reconstruction (green channel) was performed with a detail of 0.4 μm and a threshold setting of 0.5, and subsequently, a mask that contains the voxels outside of this surface was used for analysis. The axon surface analysis (red channel) was performed with a detail of 0.2 μm and a threshold setting of 5, and a mask of this was created for analysis. The voxels outside the surface mask of the green channel were colocalized with the surface of the red channel mask, adjusting the threshold to a value of 2 for all images. The colocalization mask was analyzed with the spot detection tool, detecting particles between 80 and 400 nm. The particle density was determined by taking the number of spots and dividing for the axonal volume. Scale bar, 20 μm. [file Image_1.tiff]

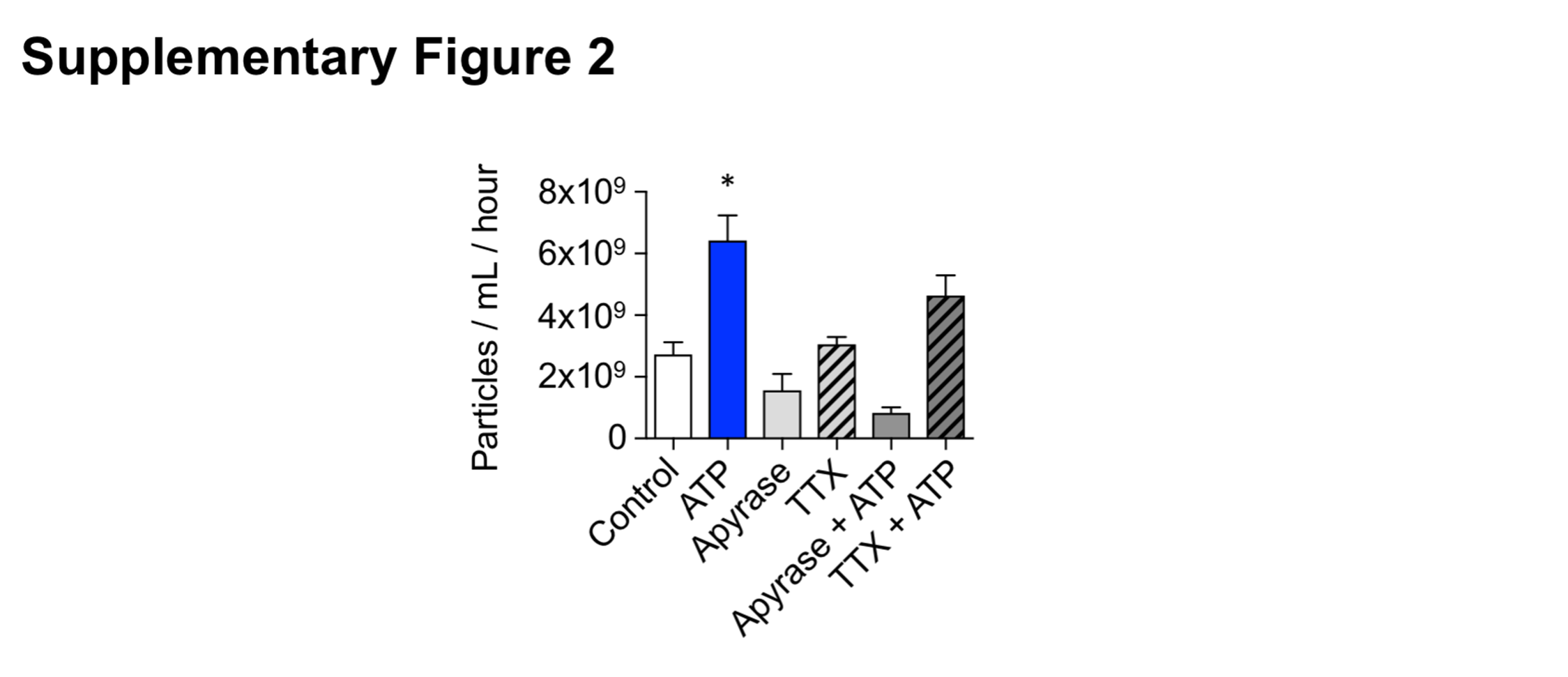

Supplement: Supplementary Figure 2 — TTX and apyrase treatments do not affect exosome release from rSC. rSCs were treated with ATP, TTX, and/or apyrase, and the conditioned media from each condition was centrifuged to purify sEVs. Samples were measured by NTA to analyze the release of particles in 1 h. In all quantifications, average and SEM values of at least three independent experiments are shown (*p < 0.05). [file Image_2.tiff]

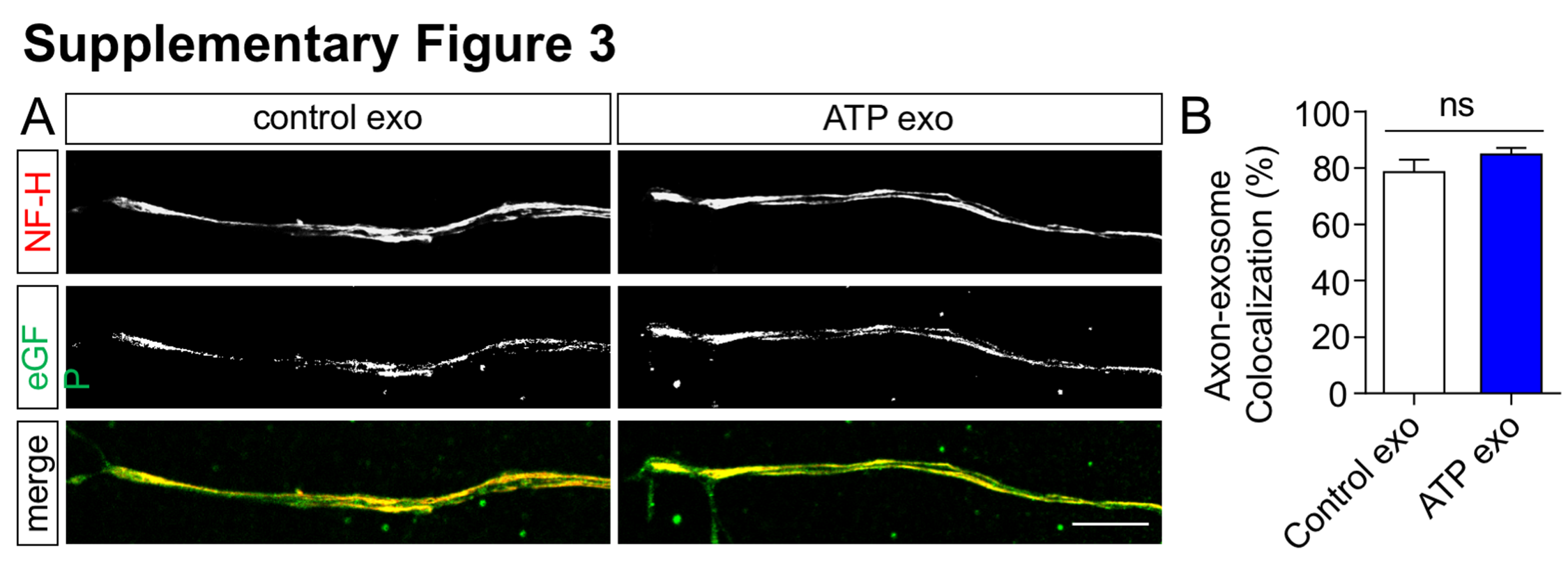

Supplement: Supplementary Figure 3 — Internalization of rSC exosomes by DRG neurons. Exosomes were isolated from control or ATP-treated rSCs transfected with CMV-palm-eGFP to visualize EVs. (A) DRG explants were treated with 5 μg of EVs for 3 h, washed, and immunostained against neurofilament heavy chain (NF-H, red) and GFP (green). Scale bar, 2 μm. (B) The axon-exosome colocalization percentage was obtained from deconvolved z-stack confocal images by measuring the eGFP mean staining area colocalized with the NF-H staining. Scale bar, 15 μm. In all quantifications, average and SEM values of at least three independent experiments are shown. [file Image_3.tiff]
